# Radiologic findings as a determinant and no effect of macrolide resistance on clinical course of *Mycoplasma pneumoniae* pneumonia

**DOI:** 10.1186/s12879-017-2500-z

**Published:** 2017-06-07

**Authors:** In Ae Yoon, Ki Bae Hong, Hoan Jong Lee, Ki Wook Yun, Ji Young Park, Young Hoon Choi, Woo Sun Kim, Hyunju Lee, Byung Wook Eun, Young Min Ahn, Eun Young Cho, Hwa Jin Cho, Eun Hwa Choi

**Affiliations:** 1 Department of Pediatrics, Seoul National University Children’s Hospital, Seoul, South Korea; 2 Department of Pediatrics, Seoul National University College of Medicine, Seoul, South Korea; 3 Department of Radiology, Seoul National University Hospital, Seoul, South Korea; 4 Department of Pediatrics, Seoul National University Bundang Hospital, Seongnam, South Korea; 5 Department of Pediatrics, Eulji Hospital, Seoul, South Korea; 6 Department of Pediatrics, Chungnam National University Hospital, Daejeon, South Korea; 7 Department of Pediatrics, Chonnam National University Hospital, Gwangju, South Korea

**Keywords:** *Mycoplasma pneumoniae*, Pneumonia, Macrolides, Resistance, Radiologic findings

## Abstract

**Background:**

With the emergence of macrolide resistance, concerns about the efficacy of macrolides for the treatment of *Mycoplasma pneumoniae* (MP) pneumonia in children have been raised. This study aimed to determine the effect of macrolide resistance on the outcome of children who were hospitalized with MP pneumonia.

**Methods:**

Between 2010 and 2015, we performed culture of MP from nasopharyngeal samples obtained from children who were hospitalized with pneumonia at five hospitals in Korea. Macrolide resistance was determined by the analysis of 23S rRNA gene transition and the minimal inhibitory concentrations of four macrolides. Medical records were reviewed to analyze the clinical response to treatment with macrolides.

**Results:**

MP was detected in 116 (4.8%) of the 2436 children with pneumonia. MP pneumonia was prevalent in 2011 and 2015. Of the 116 patients with MP pneumonia, 82 (70.7%) were macrolide-resistant. There were no differences in the age distribution, total duration of fever, and chest x-ray patterns between the macrolide-susceptible and macrolide-resistant groups. After macrolide initiation, mean days to defervescence were longer in the macrolide-resistant group than in macrolide-susceptible group (5.7 days vs. 4.1 days, *P* = 0.021). However, logistic regression analysis revealed that the presence of extrapulmonary signs (*P* = 0.039), homogeneous lobar consolidation (*P* = 0.004), or parapneumonic effusion (*P <* 0.001) were associated with fever duration of ≥7 days after the initiation of macrolides, regardless of macrolide resistance.

**Conclusions:**

This study demonstrated that fever duration in MP pneumonia was determined by the radiologic findings of chest x-ray, not by the presence of macrolide resistance. The results highlight the need for future studies to assess therapeutic benefit from macrolides in the treatment of children with MP pneumonia.

## Background

*Mycoplasma pneumoniae* (MP) is one of the most common causes of community-acquired pneumonia in children and young adults, accounting for approximately 10–30% of all cases of community-acquired pneumonia [1, 2]. Outbreaks have been occurring in cycles every 3–4 years in Korea, mostly during the late summer through the early autumn [3–5]. Emerging resistance to macrolides among MP is of great concern since a macrolide-resistant MP strain was first reported in 1997 [6, 7]. The macrolide resistance rate in Japan increased from 30% in 2006 to 93% in 2011 [7]. Recent reports showed that the resistant rate was as high as 97% among children with MP pneumonia in China in 2012 [8–10]. In Korea, the macrolide resistance rate among children with MP pneumonia increased from 0% in 2000 to 62.9% in 2011 [4].

Although macrolides are recommended for the first-line treatment for MP pneumonia, the efficacy of macrolides in the treatment of *M. pneumoniae* infection remains unclear. In addition, with the increase in macrolide resistance, concerns about the efficacy of macrolides for the treatment of MP pneumonia in children have been raised [11]. In contrast, previous studies also show clinical improvement with macrolides in patients with macrolide-resistant MP infection [12]. Overall, data are limited on the clinical response to macrolides in macrolide-resistant MP pneumonia. The aim of this study was to compare the clinical response of the children hospitalized with macrolide-resistant vs. macrolide-susceptible MP pneumonia to macrolide treatment. In addition, this study sought risk factors for pronged fever in the children with MP pneumonia.

## Methods

### Subjects

Between September 2010 and December 2015, five hospitals participated in this study, including Seoul National University Children’s Hospital (Seoul), Seoul National University Bundang Hospital (Seongnam), Eulji Hospital (Seoul), Chungnam University Hospital (Daejeon), and Chonnam University Hospital (Gwangju). Cases from three hospitals were included from June 2014 to December 2015 to collect pneumonia cases from three different regions distant from Seoul, while those from Seoul National University Children’s Hospital and Eulji Hospital were enrolled throughout the study period.

This study enrolled the children who were hospitalized with a confirmed diagnosis of MP pneumonia and also were treated with macrolides without introducing or changing with tetracyclines or fluoroquinolones. Nasopharyngeal aspirates or swabs were obtained from children hospitalized with acute lower respiratory tract infections (LRTIs) as a strategy of routine patient care. Diagnosis of MP pneumonia was based on 1) the presence of infiltration in chest radiography together with clinical symptoms and signs such as fever (defined as ≥38 °C by axillary temperature), cough, or sputum and 2) MP detected by culture or positive test results for the MP *P1* gene by PCR. MP-specific antibody was measured using a microparticle agglutination test Serodia-MycoII kit (Fujirebio, Tokyo, Japan). A 4-fold or greater rise in titers or a single titer of ≥1: 640 were considered to indicate current MP infection [5]. The following conditions were excluding criteria: underlying diseases that can modify the clinical course of MP pneumonia, hospital-acquired pneumonia, bacterial coinfection, and treatment with other anti-mycoplasmal antibiotics such as levofloxacin or tetracycline.

Nasopharyngeal samples were kept at 4 °C and sent to the laboratory of the Seoul National University Children’s Hospital for the culture of MP and analysis of macrolide resistance within 24 h from the collection of the samples.

### *Mycoplasma pneumoniae* detection

Cultivation of MP was performed at the Seoul National University Children’s Hospital. Reference strain M129 (ATCC 29342) was cultured in parallel with the clinical samples using pleuropneumonia-like organism (PPLO) broth and agar. Two hundred microliters of the nasopharyngeal specimen were serially diluted 64-fold. The broth medium was composed of 70 mL of PPLO broth, 20 mL of horse serum, 10 mL of 25% yeast extract, 2.5 mL of 20% glucose, 200 μL of 1% phenol red, 1 mL of 2.5% thallium acetate, 0.5 mL of 200,000 units/mL penicillin G potassium, and 0.5 mL of 20,000 μg/mL cefotaxime. The agar was prepared with the same components as the broth medium except that cefotaxime was omitted and 1.2% agar powder was added instead of broth powder. The broth and the agar media were incubated aerobically at 37 °C for 6 weeks.

The plates were observed daily to identify color changes in the broth medium from red to transparent orange. Upon color change, 10 μL were sub-cultured onto agar plates. Spherical MP colonies were observed under a microscope at 100X magnification. In addition, *P1* gene was amplified by PCR for the confirmation of MP. DNA was also extracted directly from the nasopharyngeal samples and subsequently amplified for *P1* gene. Each reaction mixture was prepared with the following: 2 μL of template, 2 μL of 10X reaction buffer, 2.0 mM of MgCl_2_, 0.2 mM of dNTP mix, 4 pmoL of each forward and reverse primer, 1 U of Taq DNA polymerase (Takara Bio Inc., Shiga, Japan), and distilled water to a final volume of 20 μL. Reaction conditions were set as follows: denaturation at 95 °C for 30 s, 35 cycles of annealing at 58 °C, and elongation at 72 °C for 40 s. Cases were included when cultures grew MP that was subsequently confirmed to be positive for the *P1* gene by PCR. Cases with positive MP PCR of the nasopharyngeal aspirates were also included.

### Determination of macrolide resistance

PCR to amplify domain V of the 23S rRNA gene was performed on cultured MP isolates or DNA extracted from nasopharyngeal samples. The primers used were MP23SV-F (5′-TAA CTA TAA CGG TCC TAA GG) and MP23SV-R (5′-ACA CTT AGA TGC TTT CAG CG). DNA from the reference strain M129 (ATCC 29342), was used as a positive control, and distilled water was used as a negative control. The 851- bp PCR products were purified using an AccuPrep® PCR Purification Kit (Bioneer, Inc., Daejeon, Korea), and samples were sequenced to identify the transitions in domain V of the 23S rRNA gene that have been associated with macrolide resistance [13]. The presence of 23S rRNA gene transitions were regarded as resistant to macrolides. For 56 cultured strains, the minimal inhibitory concentrations (MICs) were determined by use of a microdilution method. Briefly, frozen stocks were thawed and diluted in a broth media to a concentration of 10^4^ color change units (CCU)/mL. The MICs of MP strains were then measured using microdilution method in triplicate for the following 4 antimicrobial agents: azithromycin, clarithromycin, erythromycin, and roxithromycin. Each antimicrobial was serially diluted 2-fold, with a range from 0.0008 μg/mL to 128 μg/mL. MIC was defined as the lowest antibiotic concentration at which color did not change at the time when the color of positive control (media containing MP strains only) changed [14]. If negative control (media only) showed color change, the test was discarded. MIC of the reference strain M129 was determined as a control.

### Clinical data collection and assessment of clinical outcome

**Fig. 1:**
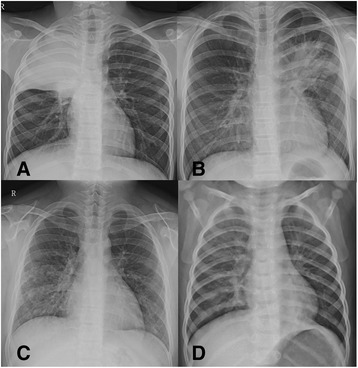
Four categories of chest x-rays of *Mycoplasma pneumoniae* pneumonia. Homogeneous dense lobar consolidation (**a**), patchy consolidation (**b**), nodular opacity (**c**), and bilateral parahilar infiltration (**d**)

The patients’ demographics, clinical manifestations, number of febrile days, duration of macrolide treatment, and the laboratory results were collected on a standardized form through review of the medical records by a pediatrician in each hospital. A febrile day was defined as a day on which the body temperature exceeded 38 °C at least once. Clinical parameters such as oxygen requirement, mechanical ventilator support were reviewed. Chest x-rays at the time of admission were reviewed independently by two radiologists. Chest x-ray findings were categorized into homogeneous dense lobar consolidation, patchy consolidation, nodular opacities, and bilateral parahilar infiltration (Fig. 1). Parapneumonic effusion, defined as ≥1 cm width on the decubitus view, was also included in the analysis. The pediatricians and radiologists were all blinded to the results of the macrolide susceptibility of MP. In the five participating hospitals, macrolide agents were initially chosen for the treatment of MP pneumonia. All physicians were unware of the results of 23S rRNA mutation during the treatment. The results from the susceptibility test did not have any impact on the choice of antibiotics because the susceptibility results take up to 6 weeks.

### Statistical analyses

The data were analyzed using SPSS software version 19.0 (SPSS Inc., Chicago, IL, USA). For all of the statistical tests, a *p*-value of <0.05 was considered statistically significant. Differences between categorical variables were tested using the chi-squared or Fisher’s exact test with univariate and multivariate logistic regression analysis. The Mann-Whitney *U* test or t-test were used to compare the age and duration of febrile days between the groups as appropriate.

## Results

### Demographics of patients with MP pneumonia

Between September 2010 and December 2015, a total of 2436 nasopharyngeal samples were obtained from children hospitalized with acute LRTIs. Of those, MP was detected in 250 samples (10.3%). A total of 116 children who were hospitalized with MP pneumonia were included after excluding cases that had any of the exclusion criteria such as underlying diseases, hospital-acquired pneumonia, bacterial coinfection, use of quinolone or tetracycline. Overall, 50.9% of the patients were male, and the mean age was 6.0 years; 9.5% were <2 years old, 31.9% were 2–4 years old, and 58.6% were ≥5 years old. The mean duration of fever was nine days (range 1–30 days), and the mean days of admission were 9 days. MP-specific antibody titers were ≥4-fold or greater in 52 (44.8%) patients and single antibody titers ≥1:640 in 49 (42.2%) patients. Remaining 15 patients did not have paired serum samples.

### Prevalence of macrolide resistance

**Fig. 2:**
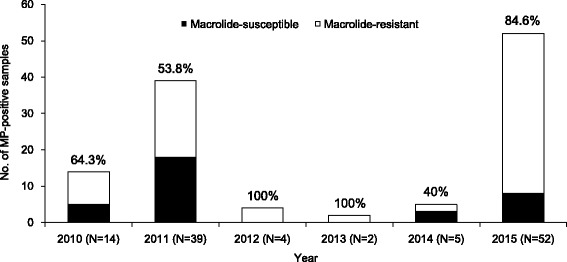
Yearly occurrence of *Mycoplasma pneumoniae* pneumonia and macrolide resistance rate in hospitalized children, 2010–2015. Macrolide resistance rates are shown on the bars for that year

The annual distribution of 116 MP-positive samples over 6 years is shown in Fig. 2. During the study period, MP pneumonia was prevalent in 2011 and in 2015. Overall, 82 samples (70.7%) carried transitions in the 23S rRNA gene. The transition observed in the 23S rRNA gene was exclusively the A2063G transition. The macrolide resistance rates during the two outbreaks were 53.8% (21/39) in 2011 and 84.6% (44/52) in 2015. Of the 59 MP isolates that underwent MIC testing for four macrolides (azithromycin, clarithromycin, erythromycin, and roxithromycin), the MICs of 23 MP isolates that lacked the transition in the 23S rRNA gene ranged from 0.001 to 0.008 μg/mL. In contrast, the MICs of the 36 MP isolates that had a transition in the 23S rRNA gene were significantly higher, ranging from 2 to >128 μg/mL (Table 1).

**Table 1:** Minimal inhibitory concentrations (MICs) to macrolides for *Mycoplasma pneumoniae* strains

Macrolides	MICs (μg/mL) for strains					
	With 23S rRNA transition (*n* = 36)			Without 23S rRNA transition (*n* = 23)		
	Range	MIC_50_	MIC_90_	Range	MIC_50_	MIC_90_
Azithromycin	1 ~ 64	8	16	0.001 ~ 0.001	0.001	0.001
Clarithromycin	8~ > 128	64	128	0.001 ~ 0.002	0.001	0.002
Erythromycin	2~ > 128	16	128	0.001 ~ 0.004	0.001	0.002
Roxithromycin	0.032 ~ 128	8	32	0.001 ~ 0.008	0.001	0.004

MIC_50_ and MIC_90_ are MICs at which 50% and 90% of the isolates were inhibited by the antimicrobials, respectively

### Clinical features of MP pneumonia

The clinical parameters and demographics of the children hospitalized with MP pneumonia were compared according to the presence of macrolide resistance (Table 2). Of the macrolides used, roxithromycin was predominantly used (*n* = 56), followed by clarithromycin (*n* = 30). One patient used azithromycin only, and 29 cases used two different macrolides during their course of treatment. There were no differences in the type of macrolides used or the mean days of treatment duration (12 days vs. 11 days) between the macrolide-susceptible and macrolide-resistant groups. The mean ages were not different between the macrolide-susceptible and macrolide-resistant groups (5.7 years vs. 6.1 years, *P* = 0.574).

**Table 2:** Demographics and clinical characteristics of *Mycoplasma pneumoniae* pneumonia to the presence of macrolide resistance

Parameters	Macrolide-susceptible (*N* = 34)	Macrolide-resistant (*N* = 82)	*P*
Male gender	22 (64.7)	37 (45.1)	0.055
Age			
Mean, years ± SD	5.7 ± 3.9	6.1 ± 3.3	0.574
< 2 years	6 (17.6)	6 (7.3)	0.104
≥ 2–4 years	12 (35.3)	25 (30.5)	
≥ 5 years	16 (47.1)	51 (62.2)	
Mean febrile days, SD	8.9 ± 3.9	9.4 ± 5.3	0.322
Fever days before initiation of macrolides			
Mean ± SD, days	4.9 ± 3.5	4.3 ± 2.6	0.315
Fever days after initiation of macrolides			
Mean ± SD, days	4.1 ± 2.9	5.7 ± 4.7	0.021
≥ 7 days	6 (17.6)	31 (37.8)	0.034
Oxygen requirement	5 (14.7)	15 (18.3)	0.217
Mechanical ventilator	0 (0)	2 (2.4)	1.0
Extrapulmonary signs	6 (17.6)	9 (11.0)	0.798
Skin rash	4 (11.8)	8 (9.8)	0.745
Hematology	1 (2.9)	1 (1.2)	0.502
Neurologic	1 (2.9)	0 (0)	0.293
Radiology findings			
Homogeneous dense lobar consolidation	9 (26.5)	35 (42.7)	0.101
Patchy consolidation	13 (38.2)	24 (29.3)	0.346
Nodular opacities	7 (20.6)	12 (14.6)	0.430
Bilateral parahilar infiltration	5 (14.7)	11 (13.4)	0.855
Parapneumonic effusion	4 (11.8)	18 (22.0)	0.298

Data are no. (%) of patients, unless otherwise indicated

Overall, the mean days of fever were nine days. There was no significant difference in the total duration of fever between the macrolide-susceptible and macrolide-resistant groups (9.4 days vs. 8.9 days, *P* = 0.322). Most patients were tested for the detection of MP no later than 7 days except 14 (12%) patients who presented after 7 days of fever. While the number of febrile days before macrolide treatment did not differ between the groups (4.9 days vs. 4.3 days, *P* = 0.315), the mean days to defervescence after macrolide initiation were longer in the macrolide-resistant group than in macrolide-susceptible group (5.7 days vs. 4.1 days, *P* = 0.021). After the initiation of macrolide treatment, more cases had a fever for more than 7 days in the macrolide-resistant group compared with those in the macrolide-susceptible group (37.8% vs. 17.6%, *P* = 0.034). There were no differences in oxygen requirement, mechanical ventilator support between the groups. Significant differences were not observed between the two groups in the presence of extrapulmonary signs.

### Relationship between clinical course and findings of chest x-ray

In comparing the findings of chest x-rays at the time of admission, the patients exhibited bilateral parahilar infiltration (*n* = 16), nodular opacity (*n* = 19), patchy consolidation (*n* = 37), and homogeneous dense lobar consolidation (*n* = 44). Parapneumonic effusion was observed in 22 patients. Overall, there was no significant difference in the proportions of specific chest x-ray patterns according to the presence of macrolide resistance (Table 2).

Mean days to defervescence following macrolide initiation were longer in children with MP pneumonia when they showed homogeneous dense consolidation (7.27 days vs. 4.01 days, *P* < 0.001) and parapneumonic effusion (9.73 days vs. 4.20 days, *P* < 0.001) than those who did not. In contrast, children with parahilar infiltration showed shorter duration of fever (1.63 days vs. 5.83 days, *P* < 0.001). Parapneumonic effusion was frequently accompanied by homogeneous dense lobar consolidation (*n* = 18) and patchy consolidation (*n* = 4). It is also notable that patients with parapneumonic effusion tended to show extrapulmonary signs more frequently than those without (77.3% vs.11.7%, *P* < 0.001).

### Risk factors associated with prolonged fever in MP pneumonia

To evaluate the factors associated with a longer fever duration, the patients were grouped by fever duration into groups of <7 days or ≥7 days (Table 3). Univariate analysis revealed that macrolide resistance (*P* = 0.034), extrapulmonary signs (*P* = 0.006), homogeneous lobar consolidation (*P* < 0.001), and parapneumonic effusion (*P* < 0.001) were significantly associated with the children with fever of 7 days or longer. In contrast, parahilar infiltration was associated with shorter fever days (*P* = 0.003).

**Table 3:** Risk factors for prolonged fever following macrolide treatment

Parameters	Fever duration following macrolide treatment		Univariate model	Multivariate model		
	<7 days (*N* = 79)	≥7 days (*N* = 37)	*P*	aOR^a^	*P*	95% CI
Male gender	40 (50.6)	19 (51.4)	0.942			
Age in years, mean ± SD	5.8 ± 3.2	6.3 ± 3.9	0.499			
Macrolide resistance	51 (64.6)	31 (83.8)	0.034	2.687	0.072	0.917–7.878
Extrapulmonary signs	9 (11.4)	12 (32.4)	0.006	3.037	0.039	1.057–8.726
Radiology findings						
Lobar consolidation	21 (26.6)	23 (62.2)	0.000	3.610	0.004	1.519–8.580
Patchy consolidation	27 (34.2)	10 (27.0)	0.441			
Nodular opacities	15 (19.0)	4 (10.8)	0.420			
Parahilar infiltration	16 (20.3)	0 (0)	0.003			
Parapneumonic effusion	5 (6.3)	17 (45.9)	0.000	9.705	0.000	3.031–31.075

Data are no. (%) of patients, unless otherwise indicated

^a^Adjusted odds ratio

Multivariate analysis showed that extrapulmonary signs (aOR; 3.037, 95% CI; 1.057–8.726), homogeneous lobar consolidation (aOR; 3.610, 95% CI; 1.519–8.580), and parapneumonic effusion (aOR; 9.705, 95% CI; 3.031–31.075) were responsible for prolonged fever ≥7 days following macrolide treatment regardless of macrolide resistance.

## Discussion

Due to increasing recognition of macrolide-resistant MP worldwide, there is a growing concern over the efficacy of macrolide treatment for children with macrolide-resistant MP pneumonia. Given the versatile features of MP pneumonia, which are determined by the patient’s age, the immunologic response of the host, and extrapulmonary manifestations [2], a more comprehensive approach must be established to analyze the clinical outcome of MP pneumonia according to the presence of macrolide resistance. In this study, we compared fever duration following macrolide treatment between the macrolide-susceptible and macrolide-resistant groups for the children hospitalized with MP pneumonia who had fever and radiological abnormalities on chest x-rays. The main findings from this study were that children with MP pneumonia tended to have prolonged fever when they accompanied extrapulmonary signs, homogeneous lobar consolidation, or parapneumonic effusion. It is important to note that macrolide resistance alone did not contribute to fever duration after the initiation of macrolide treatment.

MP containing transitions in the 23S rRNA gene has been reported worldwide since 2000. Japan reported more than 40% MP resistance during 2008–2010 [13, 15]. For China, more than 80% of MP infections are caused by macrolide-resistant strains [8, 16, 17]. Studies in the United States, Israel and Italy reported an approximately 26–30% prevalence of macrolide resistance [18–20]. Studies have demonstrated that the A2063G and A2064G transitions in domain V of 23S rRNA confer high-level resistance to 14-, 15-, 16-membered ring macrolides [21–23]. In our study, 70.7% of the patients with MP pneumonia had a 23S rRNA gene transition. Because a previous study reported that the macrolide resistance rate among children with MP pneumonia has been increasing and was 62.9% in 2011 [4], some physicians often change their treatment to levofloxacin when patients are not responding to macrolides. Thus, 34 patients (39.5%) of the 86 patients with MP pneumonia in 2015 received levofloxacin for their treatment and had to be excluded from the current study.

Most MP infections in children are known as mild and self-limiting, while only a small portion of the patients need hospitalization. The duration of symptoms can be shortened by the early administration of macrolides [11]. Initially, treatment of MP pneumonia with antimicrobials was supported by a randomized trial of 290 marine recruits that showed a shortening of fever duration, alleviation of cough, and improvement of chest x-rays [24]. An in vitro study also favored antimicrobials for the eradication of MP [25]. Before the emergence of macrolide-resistant MP, a retrospective study found that empirical therapy with macrolides decreased the duration of hospital stays, but this study had a major limitation in that microbiologic diagnosis was not adequately performed [26]. A recent systematic review that evaluated the effect of treating MP pneumonia demonstrated that there was no significant clinical benefit of antimicrobial therapy in children with MP pneumonia [27].

However, some studies have reported that patients infected with MP-resistant strains had more febrile days and a longer duration of persistent cough than those infected with MP-susceptible strains [28–30]. One study found that 15 out of 21 patients with macrolide-resistant MP pneumonia remained febrile for more than 48 h after the initiation of macrolide, but when treatment was changed to minocycline, fever disappeared within 48 h in all patients [28]. A study performed in Japan concluded that 5- to 7-day treatment with minocycline or doxycycline was effective for macrolide-resistant MP infection, as shown by the reduced number of MP DNA copies 3 days after treatment [31]. In this study, the authors also found that tosufloxacin, a fluoroquinolone, was superior to azithromycin or clarithromycin in reducing fever within 48 h after antibiotic administration for macrolide-resistant MP pneumonia. According to these studies, macrolide resistance contributes to clinical severity, and macrolides are considered as inappropriate first-choice drugs; thus, alternative treatment seem to be necessary for macrolide-resistant MP infections. In the meanwhile, a recent systematic review raised a question about benefit from macrolide treatment for MP pneumonia [32].

Our study found that macrolide resistance alone did not contribute to fever duration after macrolide treatment in children who were hospitalized for MP pneumonia. The findings may further suggest that macrolide treatment for macrolide-susceptible MP pneumonia may not contribute to significant clinical improvement compared to no antimicrobial treatment. Focal reticulonodular infiltration or perihilar interstitial infiltration are considered to be common findings of MP pneumonia [2, 33]. However, children who did not respond to macrolides more likely to show homogeneous dense lobar consolidation, extrapulmonary manifestations, and parapneumonic effusion.

This study has several limitations. Retrospective nature of the study may be subject to the possibility of incomplete clinical information. However, this study used fever as an objective parameter for clinical outcome that was measured and recorded by hospital staff. In addition, because this study assessed the clinical efficacy of macrolide for the children who were hospitalized with MP pneumonia, it may represent severe cases of MP pneumonia and cannot be generalized to overall pictures of MP pneumonia. In this study, roxithromycin was the frequently prescribed macrolide. Because high antibiotic resistance rate has been a major problem among children in Korea, there is a consensus to avoid macrolides with a long half-life such as azithromycin and clarithromycin. Despite these limitations, clinical information was gathered by those who were blinded to the results of macrolide resistance. We applied strict diagnostic criteria for MP infection, positive culture or PCR along with MP-specific antibody response, to avoid variability in diagnostic criteria. This study enrolled previously healthy children to minimize confounding factors that can contribute to clinical outcome. By excluding mild cases with MP pneumonia for whom the efficacy of antimicrobial treatment cannot often be distinguished from spontaneous resolution, clinical benefits of macrolide treatment were better assessed for those with severe manifestations. In addition, this study was first to use four distinct patterns of chest x-rays as a tool to measure severity of MP pneumonia.

## Conclusions

Given the current trend of the increasing prevalence of macrolide resistance, our study suggests that lobar consolidation with or without parapneumonic effusion and extrapulmonary manifestations were associated with severe clinical outcome of MP pneumonia while macrolide resistance was not a determinant of clinical course of MP pneumonia.
